# Supplementary material for: Evaluation of a disability-inclusive ultra-poor graduation programme in Uganda: study protocol for a cluster-randomised controlled trial with process evaluation
Source: Trials. 2024 Mar 21;25:206. doi: 10.1186/s13063-024-08040-w (PMC10956226; doi:10.1186/s13063-024-08040-w)
Supplement: Supplementary file 1 — Supplementary Material 1. [file 13063_2024_8040_MOESM1_ESM.pdf]

Written Consent Form for Impact Evaluation Survey

(For adults who are 18 and above years of age)

***Title of research study*: Evaluating the Impact of Ultra-Poor Poverty Graduation Programme in Northern Uganda**

Dear Participant,

BRAC Uganda along with London School of Hygiene and Tropical Medicine (LSHTM), is conducting a study to examine the impact of the Ultra-Poor Poverty Graduation Programme for people with and without disabilities in Kiryandongo, Gulu, and Oyam districts of Uganda. The study is funded by Department for International Development and Comic Relief. The details of lead investigators involved in this study are as follows:

Researcher	Organization	Role/Responsibility	Contact
Munshi Sulaiman	BRAC	Principal Investigator	+25[redacted]
Calum Davey	LSHTM	Principal Investigator	+44[redacted]
Christine Nabulumba	BRAC	Principal Investigator	+25[redacted]

Studies suggest that ultra-poor graduation programmes can help to move people out of poverty. These schemes include different components to achieve this goal, including providing assets to support livelihood activities, encouraging people to save money, and linking people to their benefits and entitlements. It is known that people with disabilities are more likely to be poor, and so these programmes may be useful for them. However, there is a lack of evidence of effectiveness of this intervention either in Uganda, or for people with disabilities. Thus, there is a need to assess the effectiveness of the ultra-poor graduation programme in Uganda, in particular for people with disabilities.

The purpose of this interview is to examine the impact on enrolment in the ultra-poor poverty graduation programme, in terms of improving monthly expenditures, income, socio-economic status, livelihood activities and social participation. Thus, we aim to ask you a few questions about these issues, which will help us in evaluating the results of the programme. We will interview you again in 2 years' time.

The interview will be conducted using a questionnaire and will last for about 60-90 minutes and will require answer for the questions on topics explained above. We are collecting this information from about 900 people living in Kiryandongo District, Gulu District, Nwoya and Oyam District. About half of these people interviewed will take part in the poverty graduation programme, while the others will not. Those participants who are not enrolled will allow us to compare whether or not there is a benefit or a harm to taking part in the programme.

The findings of this study will contribute in shaping the Government of Uganda's policies related to poverty alleviation activities, in particular for people with disabilities. The results will also help in guiding the donor funding. This study meets ethical standards and has been approved by Mildmay Uganda Research and Ethics Committee (MUREC) and LSHTM Ethical Committee.

This study is intended to cause no risks or discomfort to you, nor you are under any obligation to participate in this study. Your participation is completely voluntary and you can choose to not respond to any question you feel uncomfortable in answering. You can also choose to withdraw your participation in the study at any point of time during the survey. Now, it is up to you to decide whether to take part or not; choosing not to take part will not disadvantage you in any way.

In addition, the research does not include any clinical trials on you and you will not be subject to any medical treatment, if you wish to participate.

You will not be given any participation allowance for participating in this survey. However, to appreciate for your time in filling the survey we will give you a small token – a bar of soap or a kilo of sugar.

Please be sure that if you decide to participate, all the information collected through this survey will be kept confidential, anonymous and will be used only for research purposes. Any names, contact details and other business information you will provide to us will be removed from any datasets resulting from this study. We will store the data in password protected data management systems of LSHTM and BRAC. The combined data from all study participants may also be made available through an online data storage site that other people can access, but we will remove all information that may be used to identify you.

While this study won't benefit you directly, your information will be very valuable for BRAC, LSHTM and other organizations in their policy and programmes related to empowerment of people with disabilities. We will share the results of the study with you as a document, and it will also be available on a published report on internet. We will also inform you about the progress of the study. If you have any questions, any recommendation/feedback on the questions being asked or if you would like to understand more about this activity, please feel free to contact [Dr. Munshi Sulaiman on +25[redacted]].

If you have any questions about your rights as a research subject, you can call the Secretary of Mildmay Uganda Research and Ethics Committee (MUREC) (Ms. Nakubulwa Susan on [redacted]) and/or the Institutional Review Board of London School of Hygiene and Tropical Medicine (email: ethics@lshtm.ac.uk; tel: +44 20 7927 2221).

Please note that, as outlined in the participant information sheet, I may need to let a Social Protection officer to talk to you about anything which is keeping you unsafe, so I can do my best to keep you protected.

BRAC
Plot 880, Heritage Road Nsambya
PO.Box31817 (clock tower)
Kampala Uganda

T: +256 (0) 714274201
E: bracuganda@brac.net
W: www.brac.net

Incorporated in Uganda
as BRAC Uganda Limited

Evaluating the Impact of Ultra-Poor Poverty Graduation Programme in Northern Uganda

PLEASE INITIAL THE RELEVANT BOXES **Initials**

1. I confirm that I have read, and that I understand, the Participant Information Sheet. I have had the opportunity to consider the information provided, ask questions about the study, and have had these answered satisfactorily. ☐
2. I understand that my participation is voluntary and that I am free to withdraw from the survey at any time, without giving a reason. ☐
3. I consent to the questionnaire data being stored on the computers at LSHTM and BRAC. ☐
4. I consent to using my anonymised data (i.e. no names, contact details or any other information that can be used to identify me) being made available on an online data storage site that other people can access. ☐
5. I understand that if I inform the researcher that myself or someone else is at risk of harm they may have to report this to the relevant authorities - they will discuss this with me first but may be required to report with or without my permission. ☐

Signature/Thumbprint of research participant

--------------------------- --------------------------------------- ----------------

Name Signature of participant Date

Signature of the witness

--------------------------- --------------------------------------- ----------------

Name Signature of witness Date

Signature of researcher

I believe the participant is giving informed consent to participate in this study

---------------------------- -- --------------------- Name

Signature of researcher Date
