# Supplementary material for: Evaluation of a disability-inclusive ultra-poor graduation programme in Uganda: study protocol for a cluster-randomised controlled trial with process evaluation
Source: Trials. 2024 Mar 21;25:206. doi: 10.1186/s13063-024-08040-w (PMC10956226; doi:10.1186/s13063-024-08040-w)
Supplement: Supplementary file 2 — Supplementary Material 2. [file 13063_2024_8040_MOESM2_ESM.pdf]

HOUSEHOLD QUESTIONNAIRE

Household ID **(bid)**:

Cluster ID **(cid)**:

Branch ID **(bid)**:

**Section 1. Identification**				
1.1	Name of the Enumerator **(enu)**:		Enumerator ID **(enu_id)**:	
1.2	Interview Date (DD/MM/YY) **(idate)**: ___ ___ / ___ ___ / 2014		Interview Start Time **(stime)**: __ __ : __ __	
1.3	Name of Respondent **(res)**:		HH Member ID **(mid)**:	
1.4	Respondent Phone Number **(rphone)**			
1.5	Other Contact **(phone)**<br>[Include if the respondent does not have their own phone.]			
1.6	Name of Phone Owner **(powner)**:		Relationship to Respondent **(rel)**:	
1.7	If the interview cannot be taken, why not? **(why)**	1= Household not located<br>2 = Refused to participate<br>If other, specify. **(whyoth)**		

Section 2. Household Characteristics, Household Members Found in the Baseline

I would like to begin by asking you some questions about the members of your household.

NOTE TO THE INTERVIEWER: Household members are all those who live under the same roof and share food for 6 months or more.

2.1	2.2	2.3	2.4	The next questions ask about difficulties each member may have doing certain activities because of a HEALTH PROBLEM. [18- 65 years]						2.11	2.12	2.13	11.2	11.5
				**2.5**	**2.6**	**2.7**	2.8	2.9	2.10					
HM ID **(s21)**	[Where HH member name is available, verify that the name of the HH member is correct. Enter name again if not. For new members, register their full names.]	Is [NAME] male or female?<br><br>1 = Male<br>2 = Female	What is [NAME]'s age?<br><br>00 = >1 year<br>999 = Don't know	Does [NAME] have difficulty seeing, even if wearing glasses?<br>1=No difficulty<br>2=Some difficulty<br>3=A lot of difficulty<br>4=Cannot do at all	Does [NAME] have difficulty hearing, even if wearing hearing aids?<br>1=No difficulty<br>2=Some difficulty<br>3=A lot of difficulty<br>4=Cannot do at all	Does [NAME] have difficulty walking or climbing steps?<br>1=No difficulty<br>2=Some difficulty<br>3=A lot of difficulty<br>4=Cannot do at all	Does [NAME] have difficulty remembering or concentrating?<br>1=No difficulty<br>2=Some difficulty<br>3=A lot of difficulty<br>4=Cannot do at all	Does [NAME] have difficulty with self-care such as washing all over or dressing?<br>1=No difficulty<br>2=Some difficulty<br>3=A lot of difficulty<br>4=Cannot do at all	Using his/her usual language, does [NAME] have difficulty communicating, for example, understanding or being understood?<br>1=No difficulty<br>2=Some difficulty<br>3=A lot of difficulty<br>4=Cannot do at all	What is [NAME]'s marital status?<br><br>*[if older than 15]*<br><br>[SEE CODES BELOW]	Is [NAME] able to read and write a letter in any language?<br><br>1= Yes<br>0 = No	What is [NAME]'s highest level of education **completed**?<br><br>[SEE CODES BELOW]	Has [NAME] experienced an illness/injury that made him/her unable to perform normal activities for at least 5 days in the 3 months?<br>1 = Yes<br>→11.5<br>0 = No<br>99 = Don't know	[NAME] seek treatment sought at a health facility?
01	**(s22_1)**	**(s22a_1)**	**(s22b_1)**	**(s23_1)**	**(s24_1)**	**(s25_1)**				**(s26_1)**	**(s27_1)**	**(s28_1)**	**(s102_1)**	**(s105_1)**
02	**(s22_2)**	**(s22a_2)**	**(s22b_2)**	**(s23_2)**	**(s24_2)**	**(s25_2)**				**(s26_2)**	**(s27_2)**	**(s28_2)**	**(s102_2)**	**(s105_2)**
03	**(s22_3)**	**(s22a_3)**	**(s22b_3)**	**(s23_3)**	**(s24_3)**	**(s25_3)**				**(s26_3)**	**(s27_3)**	**(s28_3)**	**(s102_3)**	**(s105_3)**
04	"	"	"	"	"	"				"	"	"		
05	"	"	"	"	"	"				"	"	"		
**2.13**	**Who is the head of the household? [Write HH member ID.] (s29)**													
**2.14**	**Who is the respondent?**													
**2.15**	**Who is the primary beneficiary (index person)?**													

**CODES for 2.10**	**CODES for 2.12**
1 = Married monogamously<br>2 = Married polygamously<br>3 = Married, lives separately<br>4 = Divorced/Separated<br>5 = Widow/Widower<br>6 = Cohabiting<br>7 = Never Married	00 = No formal education<br>0 = Nursery<br>1 = P1<br>2 = P2<br>3 = P3<br>4 = P4<br>5 = P5<br>6 = P6<br>7 = P7<br>8 = S1<br>9 = S2<br>10 = S3<br>11 = S4<br>12 = S5<br>13 = S6<br>14 = Post-primary specialized training/certificate<br>15 = Post-secondary specialized training/diploma<br>16 = University degree (BA/BSc)<br>17 = Masters<br>18 = PhD<br>99 = Don't know

Section 4. Household Welfare and Assets<br>Now I would like to ask you about your home and your household resources.			
**Household Welfare**			
4.1	How many rooms for sleeping does your house have? This does not include kitchen, bathroom, etc.<br>[If the household is a single undivided room, enter "1."]		**(s31)**
4.2	What is the primary construction material of the **external wall** of the main household dwelling?	1 = Thatch, straw<br>2 = Mud and poles<br>3 = Timber<br>4 = Unburnt bricks with mud<br>5 = Burnt bricks with cement<br>6 = Cement blocks<br>96 = Other	**(s33)**
4.3	What is the primary source of drinking water for the household?	1 = Rain water<br>2 = River, stream, lake, pond, spring<br>3 = Well or bore hole<br>4 = Gravity flow scheme<br>5 = Vendor, tank truck<br>6 = Public taps<br>7 = Private connection to pipeline<br>96 = Other	**(s35)**
4.4	Does the household treat their water in any way before drinking it?	1 = Yes<br>0 = No→4.9	**(s37)**
4.5	How is the water treated?	1 = Boil<br>2 = Chemical, bleach powder, chlorine<br>3 = Filter<br>96 = Other	**(s38)**
4.6	What type of toilet does the household use?	1 = Bush (none)<br>2 = Uncovered pit latrine<br>3 = Covered pit latrine<br>4 = Flush toilet<br>96 = Other	**(s39)**
4.7	What is the primary fuel used in cooking?	1 = Firewood, saw dust<br>2 = Charcoal<br>3 = Paraffin, kerosene<br>4 = Gas<br>5 = Electricity<br>96 = Other	**(s310)**
4.8	What is the household's main lighting source?	1 = Firewood<br>2 = Tadooba<br>3 = Torch<br>4 = Paraffin, kerosene, gas lantern<br>5 = Solar<br>6 = Electricity<br>96 = Other	**(s311)**
4.9	Does every household member have at least 2 sets of clothes?	1 = Yes<br>0 = No	**(s312)**
4.10	Does every household member have a pair of shoes?	1 = Yes<br>0 = No	**(s313)**
4.11	Do you own or rent your home?	1 = Own→4.16<br>2 = Rent<br>3 = Live there for free→4.16	**(s314)**
4.16	Has the household made any home improvements – such as repairs, adding rooms, etc. – **in the last 12 months**?	1 = Yes<br>0 = No→4.19	**(s316)**
4.17	What was the improvement?	1 = New room<br>2 = New roofing<br>3 = Painting, wall improvement<br>4 = Latrine<br>5 = Plumbing<br>6 = Wiring<br>7 = Repair of damage to existing structure<br>8 = Flooring<br>9 = Plastering<br>10 = New furniture<br>96 = Other	**(s317, s317b/ s317c if many)**
4.18	What was the **actual cost** of the improvement?<br>[Enter "0" if the improvement was done with local materials and HH labour. Enter "0" if there was no payment – either in cash or in kind – for materials or labour. Estimate the value of any payment in kind.]	98 = Refused<br>99 = Don't know	**(s318)**

Household Assets			
	4.19	4.19.A	4.19.B
Line No.	[Type of Good]	How many [good] does the household own?<br><br>[If none, record "0" and → Next good.]	If you were to sell all the [good] you own today in its current condition, how much do you think you would get for it?<br><br>98 = Refused<br>99 = Don't know
01	Radio	**(s319_1a)**	**(s319_1b)**
02	Mobile phone	**(s319_2a)**	**(s319_2b)**
03	Jewellery, including beads	**(s319_3a)**	**(s319_3b)**
04	Watch/clock	"	"
05	Cassette	"	"
06	TV	"	"
07	Cooking pots		
08	Jerry cans		
09	Plastic chairs		
10	Mattresses		
11	Beds		
12	Bicycle		
13	Motorcycle/boda		
NOTE TO INTERVIEWER: Animals captured here must be animals owned exclusively by the HH, not those that the HH is watching for others or owns in partnership. Owning exclusively means that the HH could sell these animals any time they choose without consulting others outside the HH.			
14	Cow/cattle	**(s319_14a)**	**(s319_14b)**
15	Goat	**(s319_15a)**	**(s319_15b)**
16	Sheep	**(s319_16a)**	**(s319_16b)**
17	Pig	"	"
18	Chicken	"	"
19	Other animal, specify: **(s319_19oth)**	"	"
20	Hoe		
21	Panga, slashers, etc.		

Section 5. Household Consumption and Expenditures<br>Now I would like to ask you the things that your household eats and often spends money on.						
**Food and Beverage Consumption**						
**5.1**	**How many meals were eaten by HH members in the last 7 days?**					
	5.2	5.2.A	5.2.C	5.2.D	5.2.E	5.2.G
Line No.	[Type of Good]	**In the last week** has anyone in the HH eaten [food] at home or outside?<br>1 = Yes<br>0 = No<br>If no, go to the next food item.	What quantity of [food] did the HH consume **in the last week**?<br>98 = Refused<br>99 = Don't know→5.2.F	Unit<br>1 = Piece 9 = Heap<br>2 = Whole 10 = Basin<br>3 = Kilogram 11 = Pack<br>4 = Gram 12 = Sacket<br>5 = Litre 13 = Tray<br>6 = Sacks 14 = Cup<br>7 = Bunch 15 = Handful<br>8 = Teaspoon 96 = Other	How much does a [unit] of [food] cost in your nearest market?<br>98 = Refused<br>99 = Don't Know	Did you or anyone else in the household eat [food] **yesterday** during either the day or night?<br>1 = Yes<br>0 = No
01	Rice	**(s42a_1)**	**(s42c_1)**	**(s42d_1)**	**(s42e_1)**	**(s42g_1)**
02	Maize flour	**(s42a_2)**	**(s42c_2)**	**(s42d_2)**	**(s42e_2)**	**(s42g_2)**
03	Maize cobs, roasted	**(s42a_3)**	**(s42c_3)**	**(s42d_3)**	**(s42e_3)**	**(s42g_3)**
04	Millet	"	"	"	"	"
05	Sorghum	"	"	"	"	"
06	Matooke	"	"	"	"	"
07	Irish potatoes					
08	Sweet potatoes					
09	Cassava					
10	Yams					
11	Atapa or Kalo					
12	Pumpkin					
13	Carrots					
14	Cabbage					
15	Sukuma, kale, other local greens					
16	Tomatoes					
17	Onions					

	5.2	5.2.A	5.2.C	5.2.D	5.2.E	5.2.G
Line No.	[Type of Good]	**In the last week** has anyone in the HH eaten [food] at home or outside?<br><br>1 = Yes<br>0 = No<br>If no, go to the next food item.	What quantity of [food] did the HH consume **in the last week**?<br><br>98 = Refused<br>99 = Don't know→5.2.F	Unit<br><br>1 = Piece 9 = Heap<br>2 = Whole 10 = Basin<br>3 = Kilogram 11 = Pack<br>4 = Gram 12 = Sacket<br>5 = Litre 13 = Tray<br>6 = Sacks 14 = Cup<br>7 = Bunch 15 = Handful<br>8 = Teaspoon 96 = Other	How much does a [unit] of [food] cost in your nearest market?<br><br>98 = Refused<br>99 = Don't Know	Did you or anyone else in the household eat [food] **yesterday** during either the day or night?<br><br>1 = Yes<br>0 = No
18	Mango	**(s42a_1)**	**(s42c_1)**	**(s42d_1)**	**(s42e_1)**	**(s42g_1)**
19	Paw-paw	**(s42a_2)**	**(s42c_2)**	**(s42d_2)**	**(s42e_2)**	**(s42g_2)**
20	Jackfruit	**(s42a_3)**	**(s42c_3)**	**(s42d_3)**	**(s42e_3)**	**(s42g_3)**
21	Avocado	"	"	"	"	"
22	Sweet bananas	"	"	"	"	"
23	Other fruit/vegetable **(s42_23oth)**	"	"	"	"	"
24	Liver, kidney, or organ meats					
25	Beef, pork, chicken, other meat					
26	Fresh or dried fish					
27	Eggs					
28	Milk, yogurt, cream, etc.					
29	Groundnuts or simsim					
30	Beans, cowpeas or peas					
31	Cooking oil					
32	Sugar/Tea/Coffee					
36	Alcohol/local brew					
37	Other, specify: **(s42_37oth)**					

Recurrent Expenses			
	5.3	5.3.A	5.3.B
Line No.	[Type of good or service]	**In the last month** has anyone in the household spent money on [good or service]?<br><br>1 = Yes<br>0 = No→Next [good or service]	How much did your HH spend on [good or service] **in the last month**?<br><br>[in cash or in kind]<br><br>98 = Refused<br>99 = Don't know
01	Milling grains and staple crops into flour	**(s43_1a)**	**(s43_1b)**
02	Water: bottled, piped, or from a tank	**(s43_2a)**	**(s43_2b)**
03	Electricity	**(s43_3a)**	**(s43_3b)**
04	Charcoal or firewood	"	"
05	Other fuel, such as paraffin, kerosene, etc.	"	"
06	Household operation products: laundry soap, toilet paper, bar soap, dish washing paste, broom, matches, etc.	"	"
07	Cosmetics and personal toiletries: tooth paste, lotion, hair products, etc.		
08	Hair dressing, beauty, barber or salon		
09	Petrol or diesel		
10	Repairs/spare parts for machinery, bikes, boda, etc.		
11	Transportation fares		
12	Mobile air time and charging		
13	Recreation and entertainment: entry fees, photography, music, magazines, etc.		
14	Rent (check if they rent house)		
15	Other services (including household help), specify: **(s43a_15oth)**		

Infrequent Expenses			
	5.4	5.4.A	5.4.B
Line No.	[Type of good or service]	**In the last year** has anyone in the household spent money on [good or service]?<br><br>1 = Yes<br>0 = No→Next [good or service]	How much did your HH spend on [good or service] **in the last year**?<br><br>[in cash or in kind]<br><br>98 = Refused<br>99 = Don't know
01	Men's clothing	**(s44_1a)**	**(s44_1b)**
02	Women's clothing	**(s44_2a)**	**(s44_2b)**
03	Children's clothing (excluding school uniforms)	**(s44_3a)**	**(s44_3b)**
04	Men's footwear	"	"
05	Women's footwear	"	"
06	Children's footwear	"	"

	5.4	5.4.A	5.4.B
Line No.	[Type of good or service]	**In the last year** has anyone in the household spent money on [good or service]?<br><br>1 = Yes<br>0 = No→Next [good or service]	How much did your HH spend on [good or service] **in the last year**?<br><br>[in cash or in kind]<br><br>98 = Refused<br>99 = Don't know
07	Other clothing material, tailoring and sewing tools (thread, needles, buttons, etc.)	**(s44_7a)**	**(s44_7b)**
08	Furniture, mattresses, etc.	**(s44_8a)**	**(s44_8b)**
09	Textiles: bed sheets, blankets, etc.	**(s44_9a)**	**(s44_9b)**
10	Household glassware, tableware and utensils	"	"
11	Household appliances: jiko, kerosene stove, hot pot, kettle, etc.	"	"
12	Medicines: anti-worming, cold tablets (piritin), vaccines, bandages, contraceptives, malarial medication, pain killers (paracetamol), prescriptions etc.	"	"
14	Mobility aids (e.g. wheelchair, cane, crutches)(device or repairs)		
15	Other medical/assistive devices (e.g. Spectacles, contact lenses, hearing aid) (new, replacement)		
13	Doctor's consultation fees		
14	Hospital/clinic charges		
	Traditional medicine		
15	Other health expenditures, specify:<br>**(s44a_15oth)**		
16	School fees including PTA		
17	Boarding and lodging (for schooling children)		
18	School uniforms		
19	Books and supplies		
	Specialized schooling supports for children with disabilities (e.g. Braille, Sign Lanugage, communication boards)		
20	Other educational expenses, specify:<br>**(s44a_20oth)**		
21	Ceremonial expenses: wedding, funeral, etc.		
22	Fees, taxes, premiums, etc.		
23	Other, specify:<br>**(s44a_23oth)**		

**Section 6. Household Agricultural and Livestock Activities**<br>Now I would like to ask you about cultivation in the last year as a households.{both seasons combined}								
**PLANTING ACTIVITIES**								
**Perennial Crops**								
Line No.	6.1	6.1.A	6.1.B	6.1.C	6.1.D	6.1.E	6.1.F	6.1.G
		Did anyone in your household cultivate any [crop] **last one year)?**<br><br>1 = Yes<br>0 = No→Next crop.	How many acres of [crop] did your HH cultivate **last year**?<br><br>[[SEE CODES BELOW]	What quantity of [crop] did you produce **last year**?<br><br>Enter "00" if still growing *and has not produced anything at all.*→Next crop.<br><br>Enter "0" if none produced.→Next crop.	Unit<br>1 = Whole 5 = Heap<br>2 = Kg 6 = Basin<br>3 = Sack 7 = Cup<br>4 = Bunch 96 = Other	Did you sell any [crop] **last year**?<br><br>1 = Yes<br>0 = No→Next crop.	How many [units] of [crop] did you sell?	How much did you sell [crop] for in total?
	**Perennial crop**							
01	Matooke	**(sn51a_1)**	**(sn51b_1)**	**(sn51c_1)**	**(sn51d_1)**	**(sn51e_1)**	**(sn51f_1)**	**(sn51g_1)**
02	Cassava	**(sn51a_2)**	**(sn51b_2)**	**(sn51c_2)**	**(sn51d_2)**	**(sn51e_2)**	**(sn51f_2)**	**(sn51g_2)**
03	Yams	"	"	"	"	"	"	"
04	Sweet bananas	"	"	"	"	"	"	"
05	Coffee							
06	Tea							
07	Sugarcane							
08	Fruit tree, specify: **(sn51a_8oth)**							
09	Fruit tree, specify: **(sn51a_9oth)**							
	Annual Crops							
10	**See codes below**							

Codes for 6.1.B

A = One quarter of an acre	E = Three quarters of an acre	I = Two and a half acres	961 = Other less than 4 acres
B = One third of an acre	F = One acre	J = Three acres	962 = Other more than 4 acres
C = One half of an acre	G = One and a half acres	K = Three and a half acres	
D =Two thirds of an acre	H = Two acres	L = Four acres	

Codes for 6.1 Annual Crops

1 = Maize	6 = Sorghum	11 = Cow peas	16 = Cabbages	21 = Pumpkin
2 = Millet	7 = Groudnuts	12= Peas	17=Okra (lady's fingers)	22 = Pineapple
3 = Rice	8 = Sim sim	13= Kale of Sukuma wiki	18 = Onions	23 = Cotton
4 = Irish potatoes	9 = Beans	14 = Local greens	19 = Tomatoes	24 = Tobacco
5= Sweet potatoes	10 = Soya beans	15 = Eggplants	20 = Carrots	96 = Other, specify

AGRICULTURAL INPUTS			
	6.10	6.10.A	6.10.B
Line No.	[Type of Agricultural Input]	Has anyone in your HH spent anything on [input] **in the last one year?**<br>1 = Yes<br>0 = No→6.10.C	How much have you spent on [input] **the last one year**?<br>98 = Refuse<br>99 = Don't know
01	Seeds	**(s58a_1)**	**(s58b_1)**
02	Buying tools	**(s58a_2)**	**(s58b_2)**
03	Manure or compost	**(s58a_3)**	**(s58b_3)**
04	Chemical fertilizer	"	"
05	Pesticides, herbicides or fungicides	"	"
06	Hired labor	"	"
07	Renting machinery		
08	Other, such as ox plough, specify: **(s58a_8oth, s58c_8oth)**		

6.12.A	How many acres of land do the household own in total?<br>[SEE CODES BELOW]	**(s55a)**	6.12.B	What is the maximum number of acres of land that the household could cultivate on if they had the capital and the seeds, etc.?<br>[SEE CODES BELOW]	**(s55b)**
6.13	How many plots does your household typically farm on each season?	**(s56)**		

6.14	6.14.A	6.14.C	6.14.D	6.14.E	6.14.F	6.14.H	6.14.I	6.14.J
1 = Yes<br>0 = No→9.1	How many minutes does it take to walk from the home to the plot?<br>[One direction only.]<br>Enter "0" if the plot is on the homestead.	What is the size of the plot?<br>(in acres)<br>[SEE CODES BELOW]	What was the primary use of the plot in the last planting season?<br>1 = Cultivated<br>2 = Fallow<br>3 = Grazing land<br>96 = Other	Which household member primarily works on this plot?<br>[Enter HH member ID, multiple allowed]	Does the household have a title or local registration (LC1) of ownership for the plot?<br>1 = Yes→6.14.G<br>0 = No→6.14.H	Is the plot rented?<br>1= Yes→6.14.I<br>0 = No→6.14.J	How much is the rent **per season**?<br>[in UGX or estimate value in kind]	Is the plot communal/on communal land?<br>1= Yes<br>0 = No
01	**(s57a_1)**	**(s57c_1)**	**(s57d_1)**	**(s57e_1a/b/c/d, one for each HH member)**	**(s57f_1)**	**(s57h_1)**	**(s57i_1)**	**(s57j_1)**
02	**(s57a_2)**	**(s57c_2)**	**(s57d_2)**	**(s57e_2a/b/c/d)**	**(s57f_2)**	**(s57h_2)**	**(s57i_2)**	**(s57j_2)**
03	"	"	"	"	"			
04								

Codes for 6.12.A, 6.12.B, and 6.14.C

A = One quarter of an acre	C = One half of an acre	E = Three quarters of an acre	G = One and a half acres	I = Two and a half acres	K = Three and a half acres	961 = Other less than 4 acres
B = One third of an acre	D = Two thirds of an acre	F = One acre	H = Two acres	J = Three acres	L = Four acres	962 = Other more than 4 acres

Livestock Activities
NOTE TO THE INTERVIEWER: Livestock can be owned exclusively or they can be reared in partnership with another HH, even where only one household is keeping that/those animals on their property, depending on whether the household uniquely provides the inputs for rearing that animal and can uniquely choose to sell that animal. Be careful of understanding this difference and capturing animals accurately. Remember that, just like questions on cultivation, for all questions regarding partnership in rearing livestock you should record only **the HH's share**, not the overall amount.

Household Alone: All of the following questions pertain to animals your HH owns exclusively.

	6.15	6.15.A	6.15.G	6.15.I	6.15.J
Line No.	[Type of livestock]	How many [animals] do your HH have now?	**In the last year** how many [animals] did your HH consume?	**In the last year** how many [animals] did your HH sell?<br>If none, enter "0."→ Next animal.	How much did you sell each [animal] for on average?
01	Chicken	**(s59a_1)**	**(s59g_1)**	**(s59i_1)**	**(s59j_1)**
02	Sheep	**(s59a_2)**	**(s59g_2)**	**(s59i_2)**	**(s59j_2)**
03	Goat	**(s59a_3)**	**(s59g_3)**	**(s59i_3)**	**(s59j_3)**
04	Pig	"	"	"	"
05	Cow	"	"	"	"
06	Other, specify: **(s59a_6oth)**	"	"	"	"

	6.16	6.16.A	6.16.B
Line No.	[Type of Livestock Input]	Did you spend anything on [input] for animals owned exclusively **in the last year**?<br>1 = Yes 0 = No→Next livestock expenditure item.	How much money did you spend on [input] for animals owned exclusively **in the last year**?<br>98 = Refused 99 = Don't know
01	Buying live animals	**(s510a_1)**	**(s510b_1)**
02	Livestock/poultry feed	**(s510a_2)**	**(s510b_2)**
03	Veterinary services, medicines and vaccines	"	"
04	Other, such as hired labour for herding, etc., specify: **(s510a_4oth)**	"	"

	6.17	6.17.A	6.17.B	6.17.C	6.17.D	6.17.E	6.17.F	6.17.G	6.17.H
Line No.	[Type of animal product]	**In the last year** did you produce [animal product] for animals owned?<br>1 = Yes<br>0 = No→Next animal product.	What quantity of [animal product] did you produce?	Unit<br>1 = Piece<br>2 = Kilogram<br>3 = Liter<br>4 = Tray<br>96 = Other	What is the price of [animal product] per [unit] in your local market?	Did you sell any [animal product] **in the last year**?<br>1 = Yes<br>0 = No→6.17.H	How many [units] of [animal product] did you sell **in the last year**?	How much did you sell each [unit] of [animal product] for?	How many [units] of [animal product] did you consume?
01	Milk/cream	**(s511a_2)**	**(s511b_2)**	**(s511c_2)**	**(s511d_2)**	**(s511e_2)**	**(s511f_2)**	**(s511g_2)**	**(s511h_2)**
02	Butter/ghee	**(s511a_3)**	**(s511b_3)**	**(s511c_3)**	**(s511d_3)**	**(s511e_3)**	**(s511f_3)**	**(s511g_3)**	**(s511h_3)**
03	Eggs	"	"	"	"	"	"	"	"
04	Other, specify: **(s511_5oth)**								

Section 7. Other Household Income-Generating Activities

Now I would like to ask you about income-generating activities you or anyone else in your household engages in.

Household Members' Activities

Ask for all HH members *15 years and above*. Include each HH member on a separate line.

7.1	7.2	7.3	7.4	7.5	7.6	7.7	7.10	7.11	7.12	7.13	7.14
HM ID	**In the last week** did [NAME] do **more than one** income-generating activity, such as farming, digging for others, running a business, or doing a job?<br><br>1= Yes<br>0 = No	**In the last week** did [NAME] do any work for payment from somebody else (either in money or in kind), including paid agricultural labor, even if it was for only one hour?<br><br>1 = Yes→7.5<br>0 = No	Does [NAME] have any such paid work that [NAME] will definitely return to **in the next month**?<br><br>1 = Yes<br>0 = No	**In the last week** did [NAME] run a business of any size, for themself or with one or more partners, even if it was only for one hour?<br><br>1 = Yes→7.7<br>0 = No	Does [NAME] run any business that [NAME] will definitely return to **in the next month?**<br><br>1 = Yes<br>0 = No	**In the last week** did [NAME] help in any kind of business run by another HH member, even if it was only for one hour?<br><br>1 =Yes→7.9<br>0 = No	**In the last week** did [NAME] work on the HH farm or with HH livestock?<br><br>1 = Yes →7.13<br>0 = No	Will [NAME] definitely work on the HH farm or with HH livestock **in the next month**?<br><br>1 = Yes<br>0 = No	**In the last four weeks** was [NAME] looking for any kind of job?<br><br>1 = Yes<br>0 = No	**In the last four weeks** was [NAME] trying to start any kind of business?<br><br>1 = Yes<br>0 = No	What is the reason [NAME] has not worked in the last four weeks?
(hmid)	**(s62_*hmid)***	**(s63_*hmid)***	**(s64_*hmid)***	**(s65_*hmid)***	**(s66_*hmid)***	**(s67_*hmid)***	**(s611_*hmid)***	**(s612_*hmid)***	**(s613_*hmid)***	**(s614_ *hmid)***	
(hmid2)	**(s62_*hmid2)***	**(s63_*hmid2)***	**(s64_ *hmid2)***	**(s65_*hmid2)***	**(s66_ *hmid2)***	**(s67_*hmid2)***	**(s611 _*hmid2)***	**(s612 _*hmid2)***	**(s613 _*hmid2)***	**(s614_ *hmid2)***	
"	"	"	"	"	"	"	"	"	"	"	
"	"	"	"	"	"	"	"	"	"	"	

Codes for 7.14

1= Health condition or disability
2=Still in school/training
3=Family responsibilities
4=Could not find suitable work
5=Do not know how or where to seek work
6=Do not have economic need
7=Parents/spouse did not let me work
8=No reason given
9=Other

Household Income-Generating Activities, Outside of Farming and Husbandry

NOTE TO THE INTERVIEWER: Include each income-generating activity captured on the previous page with the following exceptions: Do not include apprenticeships, assistance in businesses run by other HH members, or household farming and livestock activities. Include each income-generating activity as a separate line.

7.15	7.16	7.17	7.18	7.22	7.23	7.24
Which household member does this income-generating activity?<br><br>Enter HM ID, and include each activity on a separate line.	What kind of activity is this?<br><br>[SEE CODES BELOW]	Is this work self-employed?<br><br>1 = Yes→7.19<br>0 = No	How is [NAME] paid?<br><br>1 = Salaried<br>2 = Daily or hourly wage<br>3 = Paid by the unit<br>96 = Other	When [NAME] is doing this work, what were the household's average monthly earnings from this activity?<br><br>[Estimated value in UGX]	Does [NAME] plan to continue this activity next year?<br><br>1 = Yes→Next line.<br>0 = No	If not, why not?<br><br>[SEE CODES BELOW]
**(s615_1)**	**(s616_1)**	**(s617_1)**	**(s618_1)**	**(s622_1)**	**(s623_1)**	**(s624_1)**
**(s615_2)**	**(s616_2)**	**(s617_2)**	**(s618_2)**	**(s622_2)**	**(s623_2)**	**(s624_2)**
"	"	"	"	"	"	"
"	"	"	"	"	"	"

**Codes for 7.16**		**Codes for 7.20**	**Codes for 7.21**	**Codes for 7.24**	
1 = Agricultural day labor	10 = Home-based business or small-scale trading	1 = Another household member [specify HM ID]	1 = Same LC1	1 = Not profitable	6 = Found a better opportunity
2 = Non-agri. day labor	11 = Small shop or retail	2 = Other relative(s)	2 = Different LC1, same district	2 = Illness, injury	7 = Conflict or disagreement between business members, if group business
3 = Fishing	12 = Driver (taxi, boda, etc.)	3 = Neighbor(s)	3 = Different district	3 = Household responsibilities	96 = Other, specify
4 = Food processing for sale	13 = Teacher	4 = Friend(s)	4 = Nearest city	4 = Bad for social status	
5 = Stitching/tailoring	14 = Government worker	5 = Other community member(s)	5 = Kampala	5 = Retired	
6 = Brick-making	15 = Other salaried worker, specify	96 = Other, specify	6 = Outside the country		
7 = Charcoal-making	16 = Other self-employed, specify		96 = Other		
8 = Bee-keeping	96 = Other, specify				
9 = Timber cutting					

	8.1	8.2	8.3	8.4	8.5
Line No.	Has anyone in this household taken out a loan in the last 12 months?	What was the total loan amount?	What is the total to be repaid in the end (including interest)?<br><br>[in UGX or estimated value in kind]	How many months ago did you take the loan?<br><br>Enter "00" if the loan has been repaid.→Next loan.	What is the total remaining to be repaid?<br><br>[in UGX or estimated value in kind]
01		**(s76_1)**	**(s77_1)**	**(s78_1)**	**(s79_1)**
02		**(s76_2)**	**(s77_2)**	**(s78_2)**	**(s79_2)**
03		"	"	"	"

			**(s711)**	
	8.6	8.7	8.8	8.9
Line No.	Does anyone in the household keep savings<br>1 = Yes<br>0 = No→9.1	Where do you currently keeping savings?<br><br>[SEE CODES BELOW]	How often do you deposit money there?<br><br>1 = Monthly<br>2 = Bi-weekly<br>3 = Weekly<br>96 = Other time Period	How much do you have saved there now?<br><br>98 = Refused<br>99 = Don't know
01		**(s712_1)**	**(s714_1)**	**(s715_1)**
02		**(s712_2)**	**(s714_2)**	**(s715_2)**
03		"	"	"

Codes for 8.12
1 = Saving at home
2 = With other relative outside the household
3 = With neighbor/friend
4 = With shopkeeper
5 = SACOS, ROSCAS, other savings groups
6 = NGO
7 = Bank
96 = Other

	9.1	9.1.B
Line No.	**In the past 4 weeks**, …	How often?<br>1 = Never 2=Once or twice 2 = Once a week<br>3 = Almost every day
01	…did you worry that your household would not have enough food?	**(s81b_1)**
02	…were you or any household member not able to eat the kinds of foods you preferred because of a lack of resources?	**(s81b_2)**
03	…did you or any household member have to eat a limited variety of foods due to a lack of resources?	**(s81b_3)**
04	...did you or any household member have to eat some foods that you really did not want to eat because of a lack of resources?	"
05	…did you or any household member have to eat a smaller meal than you felt you needed because there was not enough food?	"
06	…did you or any household member have to eat fewer meals in a day because there was not enough food?	"
07	…was there ever no food to eat of any kind in your household because of lack of resources to get food?	
08	…did you or any household member go to sleep at night hungry because there was not enough food?	
09	…did you or any household member go a whole day and night without eating anything because there was not enough food?	

**Household Assistance**			
	9.7	9.7.A	9.7.B
Line No.	[Type of HH assistance]	**In the past year** did your household receive [assistance]?<br>1 = Yes<br>0 = No→Next assistance type.	How much did your household receive from [assistance] **in the last year**?<br>[in UGX or estimate value if given in kind]
01	Gifts from people outside the HH	**(s87a_1)**	**(s87b_1)**
02	Remittances	**(s87a_2)**	**(s87b_2)**
03	Food aid	**(s87a_3)**	**(s87b_3)**
04	Educational support or scholarship	"	"
05	Cash transfer from the government		
	[INSERT PROGRAMME NAMES/DESCRIPTIONS]		
05	Other NGO assistance, monetary	"	"
06	Other NGO assistance, assets, and other services	"	"
07	Pension		
08	Life insurance annuity benefits		
09	Inheritance or alimony		
9.8	Did you give a loan to anyone outside of the household **in the last year**?	1 = Yes<br>0 = No→9.10	**(s88)**
9.9	How much have you given out in loans **in the last year**?	[in UGX or estimate value if given in kind]	**(s89)**
9.10	Did you send any remittances to relatives elsewhere **in the last year**?	1 = Yes<br>0 = No→9.12	**(s810)**
9.11	How much did you send in remittances **in the last year**?	[in UGX or estimate value if given in kind]	**(s811)**
9.12	Did you give any gifts to other households **in the last year**?	1 = Yes<br>0 = No→9.14	**(s812)**
9.13	How much did you give in gifts to other households **in the last year**?	[in UGX or estimate value if given in kind]	**(s813)**

	9.14	9.14.A	9.14.B
Line No.	[Type of Training]<br><br>[NOTE TO THE INTERVIEWER: Be sure that these trainings were not provided by Village Enterprise.]	Has any HH member attended [training]?<br><br>1 = Yes<br>0 = No→Next training item.	Which HH member attended [training]?<br><br>[Enter HM ID. Multiple allowed.]
01	Agricultural training or extension services, such as training in new agricultural practices or in farmer field school groups	**(s814a_1)**	**(s814b_1, s814c_1)**
02	Vocational skills training, such as trainings in tailoring, carpentry, mechanic, brick-making, etc.	**(s814a_2)**	**(s814b_2, s814c_2)**
03	Business and financial skills training	**(s814a_3)**	**(s814b_3, s814c_3)**
04	Village Savings & Loan Association (VSLA) or other savings training	"	"
05	Leadership or management training	"	"
06	Community-based organization development, including group formation, capacity building, constitution-writing, etc.	"	"

Section 13. Survey Close Information

13.1	Interview End Time **(etime)**: ☐☐ : ☐☐			
13.2	Were other people over the age of 15 present at the time of the interview?		1 = Yes<br>0 = No→[End]	**(s19)**
13.3	Who was present?<br><br>[Multiple allowed]	1 = Husband<br>2 = Other relative, male<br>3 = Other relative, female	4 = Neighbour/friend, male<br>5 = Neighbour/friend, female<br>96 = Other	**(s10a, s10b, s10c)**
13.4	Did this person help answer questions?		1 = Yes<br>0 = No	**(s111)**

INDIVIDUAL QUESTIONNAIRE

Household ID **(bid)**: ____

Cluster ID **(cid)**: ____

Branch ID **(bid)**: ____

Individual ID (PID): ____

Section 1. Identification		
1.1	Name of the Enumerator **(enu)**:	Enumerator ID **(enu_id)**:
1.2	Interview Date (DD/MM/YY) **(date)**: ____ ____ / ____ ____ / 2014	Interview Start Time **(stime)**:
1.3	Name of Index person	HH Member ID **(mid)**:
1.4	If the interview cannot be taken, why not? **(why)**	1= Household not located<br>2 = Refused to participate<br>If other, specify. **(whyoth)**
1.5	Who is the respondent?	1=Index person alone<br>2=Index person and other person<br>3=Other person who is not the index person
1.6	Why did the index person not respond on his/her own (if 1.5 → 2, 3)	1=Index person not available<br>2=Index person has severe difficulties communicating/understanding<br>3=Other

SECTION 2. WASHINGTON GROUP EXTENDED SET[1]		
**2.1**. Do you wear glasses or contact lenses?	Yes…………..1<br>No……………2	 2⇨2.3
**2.2**. Do you have difficulty seeing, even when wearing your glasses/contact lenses?<br><br>Would you say you have: no difficulty, some difficulty, a lot of difficulty or cannot do at all?	No difficulty…………..1<br>Some difficulty……….2<br>A lot of difficulty……...3<br>Cannot do at all……...4	1⇨2.4<br>2⇨2.4<br>3⇨2.4<br>4⇨2.4
**2.3**. Do you have difficulty seeing?<br><br>Would you say you have: no difficulty, some difficulty, a lot of difficulty or cannot do at all?	No difficulty…………..1<br>Some difficulty……….2<br>A lot of difficulty……...3<br>Cannot do at all……...4	
**2.4**. Do you use a hearing aid?	Yes…………..1<br>No……………2	 2⇨2.6
**2.5**. Do you have difficulty hearing, even when using your hearing aid(s)?<br><br>Would you say you have: no difficulty, some difficulty, a lot of difficulty or cannot do at all?	No difficulty…………..1<br>Some difficulty……….2<br>A lot of difficulty……...3<br>Cannot do at all……...4	1⇨2.7<br>2⇨2.7<br>3⇨2.7<br>4⇨2.7
**2.6**. Do you have difficulty hearing?<br><br>Would you say you have: no difficulty, some difficulty, a lot of difficulty or cannot do at all?	No difficulty…………..1<br>Some difficulty……….2<br>A lot of difficulty……...3<br>Cannot do at all……...4	
**2.7.** Do you use any equipment or receive help for getting around?	Yes…………..1<br>No……………2	 2⇨2.9
**2.8**. Do you have difficulty walking or climbing steps, even when using your equipment or with help?<br><br>Would you say you have: no difficulty, some difficulty, a lot of difficulty or cannot do at all?	No difficulty…………..1<br>Some difficulty……….2<br>A lot of difficulty……...3<br>Cannot do at all……...4	1⇨2.10<br>2⇨2.10<br>3⇨2.10<br>4⇨2.10
**2.9**. Do you have difficulty walking or climbing steps?<br><br>Would you say you have: no difficulty, some difficulty, a lot of difficulty or cannot do at all?	No difficulty…………..1<br>Some difficulty……….2<br>A lot of difficulty……...3<br>Cannot do at all……...4	
**2.10**. Using your usual language, do you have difficulty communicating, for example understanding or being understood?<br><br>Would you say you have: no difficulty, some difficulty, a lot of difficulty or cannot do at all?	No difficulty…………..1<br>Some difficulty……….2<br>A lot of difficulty……...3<br>Cannot do at all……...4	
**2.11**. Do you have difficulty with self-care, such as washing all over or dressing?<br><br>Would you say you have: no difficulty, some difficulty, a lot of difficulty or cannot do at all?	No difficulty…………..1<br>Some difficulty……….2<br>A lot of difficulty……...3<br>Cannot do at all……...4	
**2.12**. Do you have difficulty raising a 2 litre bottle of water or soda from waist to eye level?	No difficulty…………..1	

[1] 1. Washington Group on Disability Statistics. *Washington Group - Extended Question Set on Functioning (WG ES-F)*. 2011; Available from: http://www.washingtongroup-disability.com/wp-content/uploads/2016/01/WG_Extended_Question_Set_on_Functioning.pdf.

WOULD YOU SAY YOU HAVE: NO DIFFICULTY, SOME DIFFICULTY, A LOT OF DIFFICULTY OR CANNOT DO AT ALL?	Some difficulty……….2<br>A lot of difficulty……...3<br>Cannot do at all……...4	
**2.13**. DO YOU HAVE DIFFICULTY USING YOUR HANDS AND FINGERS, SUCH AS PICKING UP SMALL OBJECTS, FOR EXAMPLE A BUTTON OR PENCIL, OR OPENING OR CLOSING CONTAINERS OR BOTTLES?<br><br>WOULD YOU SAY YOU HAVE: NO DIFFICULTY, SOME DIFFICULTY, A LOT OF DIFFICULTY OR CANNOT DO AT ALL?	No difficulty…………..1<br>Some difficulty……….2<br>A lot of difficulty……...3<br>Cannot do at all……...4	
**2.14**. HOW OFTEN DO YOU FEEL WORRIED, NERVOUS OR ANXIOUS?<br><br>WOULD YOU SAY: DAILY, WEEKLY, MONTHLY, A FEW TIMES A YEAR OR NEVER?	Daily…………………..1<br>Weekly………………..2<br>Monthly……………….3<br>A few times a year…..4<br>Never…………………5	5⇨2.16
**2.15.** THINKING ABOUT THE LAST TIME YOU FELT WORRIED, NERVOUS OR ANXIOUS, HOW WOULD YOU DESCRIBE THE LEVEL OF THESE FEELINGS?<br><br>WOULD YOU SAY:	A little…………………1<br>A lot…………………...2<br>Somewhere between a little and a lot…………3	
**2.16**. HOW OFTEN DO YOU FEEL DEPRESSED?<br><br>WOULD YOU SAY: DAILY, WEEKLY, MONTHLY, A FEW TIMES A YEAR OR NEVER?	Daily…………………..1<br>Weekly………………..2<br>Monthly……………….3<br>A few times a year…..4<br>Never…………………5	5⇨2.18
**2.17.** THINKING ABOUT THE LAST TIME YOU FELT DEPRESSED, HOW DEPRESSED DID YOU FEEL?<br><br>WOULD YOU SAY:	A little…………………1<br>A lot…………………...2<br>Somewhere between a little and a lot…………3	
FOR PEOPLE SCREENING POSITIVE FOR DISABILITY		
2.18 AT WHAT AGE DID YOU FIRST START EXPERIENCING THESE DIFFICULTIES (ENTER AGE IN YEARS)?	___ years<br>0 if age<1<br>999 if don't know	
2.19 [FOR EACH DOMAIN OF DIFFICULTY][2] HAVE YOU EVER BEEN TO A DOCTOR/HEALTH PROFESSIONAL ABOUT THE DIFFICULTIES YOU FACE IN THIS AREA?	Yes…………..1<br>No……………2	2⇨NEXT SECTION
2.20 [FOR EACH YES TO 2.19] WHAT TYPE OF DOCTOR/HEALTH PROFESSIONAL DID YOU SEE?	Non-specialist (e.g. primary care doctor/nurse, health worker)…………..…1<br>Specialist for the area of difficulty……….2<br>Traditional healer ….3<br>Other………………...4	

[2] "A lot" or "cannot do" for any question, or for anxiety/depression, daily symptoms of an intensity described as "a lot"

Section 3: Personal Assistance, Assistive Products/Services[3]

**3.1**. Do you have someone to assist you with your day to day activities at home or outside?	Yes…………..1<br>No……………2	2⇨3.4
**3.2** Is this assistance provided by a household member or someone else?	Household member..1<br>Someone else………2	
**3.3** Is this assistance available whenever you need it?	Yes…………..1<br>No……………2	
**3.4** How much time did this person spend **yesterday** providing assistance (in hours)?	_____ hours	
**3.4** Do you need someone to assist you with your day to day activities at home or outside?	Yes…………..1<br>No……………2	
**3.5** Do you currently use any of the following?		
a) None	Yes…………..1<br>No……………2	
b) Cane or sticks	Yes…………..1<br>No……………2	
c) Crutches, axillary or elbow	Yes…………..1<br>No……………2	
d) Orthoses, lower limb, upper limb or spinal	Yes…………..1<br>No……………2	
e) Pressure relief cushions	Yes…………..1<br>No……………2	
f) Prostheses, lower limb	Yes…………..1<br>No……………2	
g) Rollators	Yes…………..1<br>No……………2	
h) Standing frame, adjustable	Yes…………..1<br>No……………2	
i) Therapeutic footwear; diabetic, neuropathic, orthopedic	Yes…………..1<br>No……………2	
j) Tricycles	Yes…………..1<br>No……………2	
k) Walking frames or walkers	Yes…………..1<br>No……………2	
l) Wheelchair	Yes…………..1<br>No……………2	
m) White cane	Yes…………..1<br>No……………2	
n) Screen reader/text to speech software	Yes…………..1<br>No……………2	
o) Communication boards	Yes…………..1<br>No……………2	
p) Braille	Yes…………..1	

[3] Adapted from WHO Model Disability Survey

	No…………..2	
q) Sign Language	Yes…………..1<br>No…………..2	
r) Other (specify)	Yes…………..1<br>No…………..2	
**3.6 [If at least 1 Yes to 3.5]** In addition to what you use, do you think you need any other assistive products. Which ones?		
a) None	Yes…………..1<br>No…………..2	
b) Cane or sticks	Yes…………..1<br>No…………..2	
c) Crutches, axillary or elbow	Yes…………..1<br>No…………..2	
d) Orthoses, lower limb, upper limb or spinal	Yes…………..1<br>No…………..2	
e) Pressure relief cushions	Yes…………..1<br>No…………..2	
f) Prostheses, lower limb	Yes…………..1<br>No…………..2	
g) Rollators	Yes…………..1<br>No…………..2	
h) Standing frame, adjustable	Yes…………..1<br>No…………..2	
i) Therapeutic footwear; diabetic, neuropathic, orthopedic	Yes…………..1<br>No…………..2	
j) Tricycles	Yes…………..1<br>No…………..2	
k) Walking frames or walkers	Yes…………..1<br>No…………..2	
l) Wheelchair	Yes…………..1<br>No…………..2	
m) Glasses, contact lenses		
n) White cane	Yes…………..1<br>No…………..2	
o) Screen reader/text to speech software	Yes…………..1<br>No…………..2	
p) Communication boards	Yes…………..1<br>No…………..2	
q) Braille	Yes…………..1<br>No…………..2	
r) Hearing aid		
s) Sign Language	Yes…………..1<br>No…………..2	
t) Other (specify)	Yes…………..1<br>No…………..2	
3.7 You told me you do not use assistive products/services. Do you think you need any of these?		
a) None	Yes…………..1<br>No…………..2	
b) Cane or sticks	Yes…………..1	

	No…………..2	
c) Crutches, axillary or elbow	Yes…………..1<br>No…………..2	
d) Orthoses, lower limb, upper limb or spinal	Yes…………..1<br>No…………..2	
e) Pressure relief cushions	Yes…………..1<br>No…………..2	
f) Prostheses, lower limb	Yes…………..1<br>No…………..2	
g) Rollators	Yes…………..1<br>No…………..2	
h) Standing frame, adjustable	Yes…………..1<br>No…………..2	
i) Therapeutic footwear; diabetic, neuropathic, orthopedic	Yes…………..1<br>No…………..2	
j) Tricycles	Yes…………..1<br>No…………..2	
k) Walking frames or walkers	Yes…………..1<br>No…………..2	
l) Wheelchair	Yes…………..1<br>No…………..2	
m) Glasses, contact lenses		
n) White cane	Yes…………..1<br>No…………..2	
o) Screen reader/text to speech software	Yes…………..1<br>No…………..2	
p) Communication boards	Yes…………..1<br>No…………..2	
q) Braille	Yes…………..1<br>No…………..2	
r) Hearing aid		
s) Sign Language	Yes…………..1<br>No…………..2	
t) Other (specify)	Yes…………..1<br>No…………..2	

Section 4: Participation[4]

I'm going to ask you some questions about your involvement in different aspects of family, social life and society. Please listen to each one and answer yes, no, sometimes, sometimes or not applicable.

Q. #	Question	Codes					Go to Q
		Yes	No	Sometimes	N/A	Don't know	
4.1	Are you consulted about making household decisions?	1	2	3	4	5	
4.2	Do you go with the family to events such as family gatherings, social events, etc?	1	2	3	4	5	
4.3	Do you feel involved and part of the household or family?	1	2	3	4	5	
4.4	Does the family involve you in conversations?	1	2	3	4	5	
4.5	CASES: Does the family help you with daily activities/tasks?	1	2	3	4	5	"No, DK, N/A" →4.7
4.6	CASES: Do you appreciate it or like the fact that you get this help?	1	2	3	4	5	
4.7	Do you/did you take part in your own traditional practices (e.g initiation ceremonies)?	1	2	3	4	5	
4.8	CASES: Are you aware of organizations for people with disabilities (DPO)?	1	2				"No" →4.10
4.9	CASES: Are you a member of a DPO?	1	2				
4.10	Do you participate in local community meetings?	1	2	3	4	5	"No" →4.12
4.11	Do you feel your voice is being heard?	1	2			5	
4.12	Did you vote in the last election?	1	2			5	**"No"→ sect 5**
4.13	CASES: Was it related to your disability that you didn't vote?	1	2			5	

[4] SINTEF Participation Scale: Eide, A., S. Neupane, and K.G. Hem, *Living conditions among people with disability in Nepal*. 2016, SINTEF

Section 5: Attitudes[5]

Now I want to ask you some questions about the attitudes of people around you. When answering these questions please tell me on a scale from 1 to 6, where 1 is not at all and 5 means completely.

Q. #	Question	Codes					
		1<br>Not at all	2	3	4	5<br>Yes, completely	98<br>Not applicable
1.	Do you have problems getting involved in society because of the attitudes of others?	1	2	3	4	5	98
2.	Do you feel that some people treat you unfairly?	1	2	3	4	5	98
3.	Do you make your own choices about your day-to-day life? For example, where you go, what to eat.	1	2	3	4	5	98
4.	Do you feel other people accept you?	1	2	3	4	5	98
5.	Do you feel that other people respect you? For example, do you feel that others value you as a person and listen to what you have to say?	1	2	3	4	5	98
6.	Do you consider yourself a burden on society?	1	2	3	4	5	98
7.	Do people around you tend to become impatient with you?	1	2	3	4	5	98
8.	Do people around you not expect much from you?	1	2	3	4	5	98
9.	Is living with dignity a problem for you because of the attitudes and actions of others?	1	2	3	4	5	98

I'm now going to ask you some questions about safety and people's behaviour towards you.

	In past 12 months…	Yes	No	Don't know/ Refused
10.	… have you ever been beaten, scolded or discriminated against by any household member or relatives?	1	2	88
11.	…have you ever been beaten, scolded, discriminated against at work or school?	1	2	88
12.	…how often did you experience prejudice or discrimination (anywhere)?	1	2	88
13.	(PEOPLE WITH DISABILITIES ONLY) how often have you experienced prejudice or discrimination because of your disability?	1	2	88

[5] Adapted from WHO Model Disability Survey; Violence questions from SINTEF

Section 5: Environmental factors[6]

I am going to ask you some general questions about your environment.

I would like to know if the environment makes it easy or hard for you to do things you need or want.

I want you to answer the following questions on a scale from 1 to 5, where 1 means very easy and 5 means very hard.

Q. #	Question	Codes						
		1<br>Not at all	2	3	4	5<br>Yes, completely	88<br>Don't know	98<br>Not applicable
5.1	Does your workplace or educational institution make it easy or hard for you to work or learn?	1	2	3	4	5	88	98
5.2	Do health facilities you need regularly make it easy or hard for you to use them?	1	2	3	4	5	88	98
5.3	Do places where you socialize and engage in community activities make it easy or hard for you to do this?	1	2	3	4	5	88	98
5.4	Do the shops, banks and post office in your neighborhood make it easy or hard for you to use them?	1	2	3	4	5	88	98
5.5	Do your regular places of worship make it easy or hard for you to worship?	1	2	3	4	5	88	98
5.6	Does the transportation you need or want to use make it easy or hard for you to use it?	1	2	3	4	5	88	98
5.7	Does your dwelling make it easy or hard for you to live there?	1	2	3	4	5	88	98
5.8	Does the toilet of your dwelling make it easy or hard for you to use it?	1	2	3	4	5	88	98
5.9	Do the temperature, terrain and climate of the place you usually live make it easy or hard for you to live there?	1	2	3	4	5	88	98
5.10	Do the lighting, noise and crowds in your surroundings make it easy or hard for you to live there?	1	2	3	4	5	88	98
5.11	Is the information you want or need available in a format that you can understand and use?	1	2	3	4	5	88	98

[6] Adapted from WHO Model Disability Survey

Section 6: Time Allocation

6.1	How many hours did you spend on domestic and household work – including cooking, cleaning, caring for children, etc. – yesterday?	(s625)
6.2	How many hours did you spend on farming, cultivating and other agricultural activities yesterday?	(s626)
6.3	How many hours did you spend on herding and caring for livestock yesterday?	(s627)
6.4	How many hours did you spend on other business or other income-generating activities last week?	(s628)
6.5	How many hours did you spend on visiting and meeting with others (including socializing with neighbours, meeting with friends at their homes or in trading center, attending wedding or other celebrations, etc.) last week?	(s629)

Section 7: Social networks

**INTERVIEWER ASK ONLY Q183 WHEN RESPONDENT HAS CHILDREN (CHECK Q 113 PAGE 4) IF NO CHILDREN RECORD 95 AND SKIP TO 184**		
7.1	How many of your children do you feel very close to?	children______<br><br>00= none of them<br>95 = Has no (living) children<br>97=Refused<br>8= Don't know
7.2	How many of these children live near to you?	00= none<br>Record the number
7.3	In general, apart from your children, how many other relatives do you have that you feel very close to? (people that you feel at ease with, can talk to about private matters, and call on for help?)	relatives______<br><br>00= none of them<br>97= Refused<br>8= Don't know
7.4	How many of these relatives live near to you?	00= none<br>Record the number
7.5	In general, how many very close friends do you have? (People that you feel at ease with, can talk to about private matters, and call on for help?)	Friends_______<br><br>00= none of them<br>97= Refused<br>98= Don't know
7.6	How many of these friends live near to you?	00= none<br>Record the number
7.7	In the last two weeks, how often have you….<br><br>1= Not at all<br>2= Once or twice<br>3= More than twice<br>4= Daily	
	a. …been out of the house/ dwelling to attend social meetings, activities, weddings, events?	

	b. …gotten out of the house/ dwelling to visit a relative, neighbour or friend?	
	c. …had visitors to your house, such as friends, neighbours, and relatives?	
	d. …attended religious services?	
7.8	Would you like to go out more often or are you satisfied with how much you get out of the house?	1= Would like to go out more often<br>2= Satisfied<br>3= Would like to go out less often
7.9	Would you like to have more visitors or are you satisfied?	1= Would like to have more visitors<br>2= Satisfied<br>3= Would like to have fewer visitors
