# Supplementary material for: Evaluation of a disability-inclusive ultra-poor graduation programme in Uganda: study protocol for a cluster-randomised controlled trial with process evaluation
Source: Trials. 2024 Mar 21;25:206. doi: 10.1186/s13063-024-08040-w (PMC10956226; doi:10.1186/s13063-024-08040-w)
Supplement: Supplementary file 3 — Supplementary Material 3. [file 13063_2024_8040_MOESM3_ESM.docx]

**Supplemental paper 3: Process-evaluation research questions**

| Objective | Domain | Research questions |
| --- | --- | --- |
| Intervention implementation | Fidelity | Did the intervention implementation deviate from the programme design?  To what extent were cash transfers/consumption support disbursed in a timely and consistent manner?  How were programme participants linked to additional support services provided by other programmes or government bodies? |
|  | Dose | What was the duration, frequency and nature of each intervention?  How did the sequencing of interventions affect service delivery?  What was the exposure to the four pillars? |
|  | Adaptations | What changes were made to the delivery of the intervention, and why? |
|  | Reach | Did DIG effectively cover all intended recipients and what factors in selection criteria and targeting methodology most impacted coverage?  What activities were undertaken to promote community engagement?  To what extent was training on disability inclusion made available to civil society staff |
| Mechanisms | Participant response | To what extent did the programme design account for the needs of ultra-poor households and participants with disabilities?  Were the assets suitable for providing sustainable livelihoods for participants, particularly participants with disabilities?  Were key barriers to participation in the sustainable livelihoods overcome (including health, attitudes, environment)?  Were linkages to other services (including health) appropriate and sufficient to meet participant needs?  Were the financial services appropriate?  Were the community mobilization activities appropriate?  Were the community coaching appropriate? |
|  | Mediators | Which components of the DIG programme were most important for changes to the outcomes? |
|  | Unintended consequences | Was there any increase in violence experienced by participants?  Did participants take leadership roles / political positions as a result of the programme?  Were there any unanticipated changes? |
| Context | Factors that shape the theory for how intervention works | How important were community attitudes in limiting people with disabilities? |
|  | Factors that affect intervention implementation and mechanisms | Did a lack of mobile phone infrastructure impede or improve timeliness of cash transfer delivery?  How were partnership arrangements executed to support the delivery of programme components? |
|  | Mechanisms that sustain status quo or enhance effects | What were the market conditions during implementation?  How have conditions changes in light of COVID? |
